# Lateral transfer and GC content of bacterial resistance genes

**DOI:** 10.3389/fmicb.2013.00041

**Published:** 2013-03-12

**Authors:** Nabil Hayek

**Affiliations:** Department of Biochemistry, Microbiology and Immunology, University of OttawaOttawa, ON, Canada

Lateral genetic transfer (LGT) is one of the ways in which microorganisms such as bacteria could move and rearrange genetic materials from and into their genomes (Ragan and Beiko, 2009). The dissemination of antibiotic resistance genes between two microorganisms or between a microorganism and the environment could occur, for example, by conjugation, transduction, and transformation (Fondi and Fani, 2010; Stokes and Gillings, 2011). Therefore, having a resistance gene or genes on a mobile element like plasmids and genomic islands, transposons, integrons, or integrative conjugative elements will facilitate their overall distribution, which will affect among other organisms, humans and more specifically their health and survival (Stokes and Gillings, 2011). This fact, made the selection for antibiotic resistance a crucial step in microorganism's selective evolution (Waksman and Woodruff, 1940). However, even though this selection is enhanced and accelerated by the use of antibiotics (Hegreness et al., 2008), the acquisition of a new genetic material is balanced by mechanisms that restrict DNA uptake (Navarre et al., 2006; Palmer and Gilmore, 2010). One of these mechanisms is the clustered regularly interspaced short palindromic repeat (CRISPR) (van der Oost et al., 2009), which was found to be negatively related to the number of acquired resistant genes in a *Enterococcus faecalis* (Palmer and Gilmore, 2010).

Moreover, a closer look at the multidrug resistance genomic regions in bacteria shows that they could be collected from different mobile genetic elements to form what is known as pathogenicity islands (Dobrindt et al., 2004; Fournier et al., 2006; Adams et al., 2008; Hall, 2010). And contrary to higher organisms, bacterial genetic material exchange occurs between close or distant relatives spanning the three domains of life (Garcia-Vallvé et al., 2000; Mallet, 2008). Based on the previous findings, characterizing the antibiotic resistome is becoming highly important in order to detect yet unknown resistance mechanisms and to develop new antibiotics that help in the fight against potential resistant organisms (D'Costa et al., 2006).

The guanine-cytosine (GC) composition of bacterial genomes is one of the genomic taxonomic markers and a genomic signature that is considered a tool for comparison between chromosomes and plasmids (Nishida, 2012), and for distinguishing genes according to their method of transfer, i.e., horizontally or vertically (Garcia-Vallvé et al., 2000). Therefore, could such a tool provide distinctive information about resistant genes and pathogenicity islands in the source-sink model of LGTs (Miura, 1962; Pulliam, 1988; Garcia-Vallvé et al., 2000; Bohlin, 2011)? In other words, could the GC content difference between the resistant gene/pathogenicity island and its genomic context play a role in the structure and function of the host bacterium, regardless if it was acquired horizontally or vertically? Finally, could the GC content of resistant genes be incorporated in the antibiotic resistome study (D'Costa et al., 2006)?

The fact that the complete genome sequences of many bacterial and archaeal species are still unavailable makes the answers to these questions incomplete and awaiting for more sequences to be finalized. However, several studies tried to tackle these issues.

For instance, Garcia-Vallvé et al. (2000) developed a statistical method to identify potential genes that were recently horizontally transferred and classified them according to their general functions. In another study Hildebrand et al. (2010), concluded that contrary to the GC to AT mutation pattern detected in the tested bacterial species, the genomic GC content is not correlated to the this mutation bias and species were able to maintain their higher than expected GC%. Similar results were obtained by other groups (Hershberg and Petrov, 2010; Nishida, 2012), who were able to highlight the tendency to preserve a higher genomic GC content in spite of the AT mutation bias. To explain their data, the authors (Hildebrand et al., 2010) suggested a potential selective pressure that controls the GC content in certain bacterial species with high GC content. In their recent article Raghavan et al. (2012), explored this concept further. They were able to show a significant positive correlation between the expression of a transferred protein-coding gene with a high GC content and bacterial fitness. Therefore, according to the authors, this indicates that a significantly better growth rate compensates for the AT mutation bias and a better fitness could be the driving factor for maintaining a higher GC content in at least the tested bacterial strains.

Based on the discussed studies, two groups of challenges could be highlighted. The first group is related to the conducted studies and the methodologies used. The second, is related to the mutual evolutionary effects between the GC content of resistance genes and pathogenicity islands on one side and the genomic GC content on the other. Regarding the first group, a recently published study (Becq et al., 2010) shows that detecting LGT using one method could be arduous and a combination of two methods is recommended to improve the sensitivity and the specificity of the applied method. In addition, most of the studies tries to identify recently transferred genetic material without following the evolution of the transferred gene in time (Garcia-Vallvé et al., 2000). In other words, genes that appear to have a GC content close to that of the bacterial genome could have been integrated into it many years ago and adapted to the average total GC content. To overcome this issue, and with the advances in sequencing techniques, it would be interesting to design an experiment, in which the possible LGTs in the bacterial genome are identified in the first step. Then expose the bacterium to mobile elements carrying resistant genes or pathogenicity islands with different GC content, after that determine which gene or island has been acquired. The last step will be to follow the evolution of the transferred determinant(s) by sequencing the genomic material in predetermined time intervals for generations. Moreover, changing the environmental factors during the experiment could allow researchers to prospectively study the influence of the various factors on the evolution/amelioration of the GC content of the transferred resistant determinant and the bacterial genome. These type of studies would permit the assessment of the bacterial behavior in conditions that mimic the ones they encountered in their natural habitat and will allow researchers to use genes that encode physiologically relevant proteins, such as resistant genes. In addition, such prospective experiments would enable us to keep the genetic material of the studied strains intact, mainly because repeated elements like CRISPRs could influence the ability of the bacterium to acquire resistant genes as mentioned above (Palmer and Gilmore, 2010). As for the second group of challenges, it will be appealing to investigate how we could benefit from our knowledge in this field and our understanding of bacterial behavior to treat superbugs. For example, since the acquisition of genes with high GC content will lead to a better growth rate (Raghavan et al., 2012), could we use the opposite scenario to slow the growth of resistant bacterial pathogens or to alter the structure and function of certain proteins of interest that are encoded on pathogenicity islands in pathogens?

It is important to note that pathogenicity islands in *Salmonella typhimurium* (SPIs), for example, have a lower GC content than the rest of the bacterial genome (Marcus et al., 2000).

Three genes that were shown to be horizontally transferred by Fondi and Fani (2010) were selected for further analysis of their GC content. These genes are *tetA, aminoglycoside transferase*, and *β-lactamase*. The National Center for Biotechnology Information (NCBI) database was used to retrieve genomic GC% and the gene sequences in FASTA format. Microorganisms carrying the resistant determinants were selected so to represent different habitats not only human pathogens. Then the GC% of the selected gene was calculated using the Endmemo software (http://www.endmemo.com/bio/gc.php). The sequence of the gene in FASTA format was used for this purpose. Start and end codons were not deleted from the sequence for all the tested genes. A *t-test* was used to determine if the GC content of the gene and its respective genomic DNA are significantly different. Table 1, represents the collected data and results from the selected genes; *tetA, aminogylcoside transferases*, and *β-lactamase*. The data shows that while the GC content of the *lactamase* and the *transferase* was not significantly different from their genomic DNA, the GC content of the selected *tetA* gene was indeed significantly different. The *p*-values were 0.43, 0.2, and 0.02, respectively. Moreover, a *t-test* of the combined results from the three genes was conducted and it indicates that the difference is not statistically different (*p* = 0.07).

**Table 1:** **The GC content of *tetA, aminoglycoside transferase*, and β-*lactamase* was retrieved from NCBI and compared to that of the respective genomic GC %**.

**Gene**	**Gene ID**	**Bacterium**	**RefSeq**	**Gene GC %**	**Genome GC %**
*tetA*	2716475	*Escherichia coli* plasmid pC15-1a	NC_005327.1	63.66	52.6
	8877592	*Klebsiella pneumoniae* plasmid pKF3-140	NC_013951.1	63.21	52.5
	7886608	*Salmonella enterica* plasmid pAM04528	NC_012693.1	62.43	51.9
	7003405	*Haemophilus influenzae* plasmid ICEhin1056	NC_011409.1	43.36	39.1
	2653967	*Serratia marcescens* plasmid R478	NC_004989.1	43.28	36.9
	4927413	*Yersinia pestis* biovar Orientalis str. IP275 pIP1202	NC_009141.1	57.63	52.9
	6002612	*Acinetobacter baumannii* AYE	NC_010410.1	63.21	39.3
	1794537	*Rhodopirellula baltica* SH 1	NC_005027.1	57.55	55.4
	3433250	*Corynebacterium jeikeium* K411	NC_007164.1	68.50	61.40
	2797858	*Listeria monocytogenes* serotype 4b str. F2365	NC_002973.6	42.30	38.00
				*p* = 0.02	
*transferase*	1238790	*Klebsiella pneumoniae* plasmid pJHCMW1	NC_003486.1	52.97	49
	13919580	*Providencia stuartii* plasmid pTC2	NC_019375.1	53.03	52.5
	9487131	*Klebsiella pneumoniae* plasmid pNL194	NC_014368.1	53.03	53.1
	9487121	*Klebsiella pneumoniae* plasmid pNL194	NC_014368.1	52.9	53.1
	1055588	*Citrobacter freundii* plasmid pCTX-M3	NC_004464.2	51.89	51
	7156160	*Escherichia coli* UMN026	NC_011751.1	58.37	50.6
	6810778	*Salmonella enterica* subsp. *enterica* serovar Paratyphi A str. AKU_12601	NC_011147.1	54.11	52.2
	6455499	*Cupriavidus taiwanensis* LMG 19424, Chr 2	NC_010530.1	70.94	67
	6928720	*Burkholderia cenocepacia* J2315, Chr 2	NC_011001.1	71.33	66.9
	2662391	*Bordetella bronchiseptica* RB50	NC_002927.3	73.45	68.1
				*p* = 0.2	
*lactamase*	11027496	*Escherichia coli* plasmid p271A	NC_015872.1	61.5	50.2
	2598288	*Staphylococcus aureus* plasmid pLW043	NC_005054.1	26.05	30.8
	1236105	*Sinorhizobium meliloti* 1021 plasmid pSymA	NC_003037.1	61.49	60.4
	8984252	*Klebsiella pneumoniae* plasmid pKpQIL	NC_014016.1	61.33	53.9
	13905334	*Escherichia coli* plasmid p838C-R1	NC_019062.1	49.36	55.5
	7991352	*Methylobacterium extorquens* AM1	NC_012808.1	70.36	68.5
	8833487	*Xenorhabdus bovienii* SS-2004	NC_013892.1	47.96	45
	2861159	*Staphylococcus aureus* subsp. aureus MRSA252	NC_002952.2	28.66	32.8
	6001677	*Acinetobacter baumannii* AYE	NC_010410.1	39.27	39.3
	3381690	*Xanthomonas campestris* pv. campestris str. 8004	NC_007086.1	65.07	65
				*p* = 0.43	

Bacterial strains were selected from different habitat. Resistance genes were encoded either on a plasmid or on a chromosome (five examples of each). The t-test shows that the GC content of the transferase and the β-lactamase genes was not statistically significant from the GC content of their respective genomic DNA, p = *0.2* and *0.43*, respectively. However, the GC content of the tetA gene was significantly different, p = *0.02*. In addition, the GC content of the three genes combined was not statistically different from the total genomic GC content p = *0.07*.

This preliminary data is not conclusive mainly because of the tested sample size. However, it demonstrates, in my opinion, the need to study the evolution of the GC content of each resistant gene individually rather than collectively. In addition, a database could be created for this purpose in order to track the development and the continuously modified sequences of these resistant genes.

In conclusion, the evolution of resistant genes and pathogenicity islands along with the bacterial genome in terms of their GC content could open the door for new ways to understand the behavior of microorganisms that could be consciously modifying their DNA dinucleotide composition under various stresses such as the presence of antibiotics. This in return will permit the application of the acquired information to more efficiently treat and live with pathogens.
